# KDM6B Negatively Regulates the Neurogenesis Potential of Apical Papilla Stem Cells via HES1

**DOI:** 10.3390/ijms241310608

**Published:** 2023-06-25

**Authors:** Chen Zhang, Weilong Ye, Mengyao Zhao, Lujue Long, Dengsheng Xia, Zhipeng Fan

**Affiliations:** 1 Laboratory of Molecular Signaling and Stem Cells Therapy, Beijing Key Laboratory of Tooth Regeneration and Function Reconstruction, Beijing Stomatological Hospital, School of Stomatology, Capital Medical University, Beijing 100050, China; chenz1991@163.com (C.Z.); yqw83268910@126.com (W.Y.); m18231581912@163.com (M.Z.); longlujue@csu.edu.cn (L.L.); 2 Department of Dental Emergency, Beijing Stomatological Hospital, School of Stomatology, Capital Medical University, Beijing 100050, China; dsxia@mail.ccmu.edu.cn; 3 Beijing Laboratory of Oral Health, Capital Medical University, Beijing 100069, China; 4 Research Unit of Tooth Development and Regeneration, Chinese Academy of Medical Sciences, Beijing 100069, China

**Keywords:** lysine (K)-specific demethylase 6B (KDM6B), neurogenesis potential, stem cells from apical papilla (SCAPs), hairy and enhancer of split 1 (HES1)

## Abstract

Stem cells from the apical papilla (SCAPs) are used to regulate the microenvironment of nerve defects. KDM6B, which functions as an H3K27me3 demethylase, is known to play a crucial role in neurogenesis. However, the mechanism by which KDM6B influences the neurogenesis potential of SCAPs remains unclear. We evaluated the expression of neural markers in SCAPs by using real-time RT-PCR and immunofluorescence staining. To assess the effectiveness of SCAP transplantation in the SCI model, we used the BBB scale to evaluate motor function. Additionally, toluidine blue staining and Immunofluorescence staining of NCAM, NEFM, β-III-tubulin, and Nestin were used to assess nerve tissue remodeling. Further analysis was conducted through Microarray analysis and ChIP assay to study the molecular mechanisms. Our results show that KDM6B inhibits the expression of NeuroD, TH, β-III tubulin, and Nestin. In vivo studies indicate that the SCAP-KDM6Bsh group is highly effective in restoring spinal cord structure and motor function in rats suffering from SCI. Our findings suggest that KDM6B directly binds to the HES1 promoter via regulating H3K27me3 and HES1 expression. In conclusion, our study can help understand the regulatory role of KDM6B in neurogenesis and provide more effective treatments for nerve injury.

## 1. Introduction

Nerve defects caused by tumors and trauma can significantly impact the motor ability and quality of life of patients. The new incidence rate is approximately 40 cases per million population [1], with an average age of over 40 years and a higher prevalence in males [2]. The cost of medical treatment for each patient, from injury to recovery, is high [3]. Although existing therapeutic means such as surgical decompression, physiotherapy, and drug support are available, they have limited efficacy due to their restricted indications and application strategies [4]. Recently, stem cell therapy has emerged as a potential treatment option that has gained popularity. By implanting exogenous stem cells into the spinal cord injury (SCI) region, these cells can play a crucial role in regulating the microenvironment of neural tissue regeneration. This includes regulating inflammation and inhibiting scar formation, among other effects [5,6]. While the stem cells will eventually degrade, the nutritional factors secreted by them and the improvement in axon extension are critical in promoting the recovery of neurons and signal transduction [7,8].

Epigenetic modifications are known to play a crucial role in neurogenesis, learning, and memory. During the process of neural repair, histone lysine methylation modifications have been shown to influence the function of repaired neural tissue by interfering with the expression of specific genes [9,10,11,12,13]. KDM6B possesses a JMJD sequence that allows it to achieve targeted demethylation modification of H3K27me3. Studies have shown that during the development of the embryonic cerebral cortex and forebrain subventricular region, KDM6B forms polymers that interfere with DLX2 expression, ultimately affecting the structure of neural tissue [14]. Inhibition of KDM6B can activate neuronal differentiation by interfering with H3K27me3, promoting the differentiation of neural stem cells into neurons and influencing the development of various brain regions [15]. KDM6B also regulates neural precursor cell development by interacting with H3K27, thereby influencing the expression of Nestin, which is a key marker of neural precursor cells [16,17]. Furthermore, KDM6B is involved in regulating the development of medial, lateral, and preganglionic motor columns [18]. In the directed neuroectodermal differentiation system of human embryonic stem cells, it has been observed that the knockout of KDM6B leads to a higher efficiency of neuroectodermal induction in human pluripotent stem cells [19]. The origin of cells used in neuro-regenerative medicine is a matter of concern, with embryonic stem cells and neural stem cells being the ideal sources as they can serve as seed cells while shaping the neural regeneration microenvironment. However, their limited accessibility and serious ethical issues make them difficult to apply in clinical practice.

SCAPs are a type of stem cell derived from neural crest cells and have the potential to differentiate into various types of tissue. Additionally, they highly express various neural markers such as brain-derived neurotrophic factor (BDNF), Glial cell line-derived Neurotrophic Factor (GDNF), Angiopoietin-1 (ANGPT1), and vascular endothelial growth factor A (VEGFA) [20]. SCAPs have demonstrated the ability to promote axon neurite outgrowth and axon regeneration through BDNF secretion [21]. Additionally, SCAPs are involved in various nerve repair behaviors, including neuroinflammatory transitions and oligodendrocyte progenitor cell development [22]. Thus, the accumulated evidence supports the notion that SCAPs have the potential to be an effective solution for addressing nerve-related injuries and disorders [23].

In our study, we investigated the role of KDM6B in neurogenesis in SCAPs. Our results suggest that the knockdown of KDM6B in SCAPs has a restorative effect on motor function in rats with SCI by enhancing the neurogenesis potential of SCAPs. These findings provide valuable insight into the potential applications of SCAPs in nerve repair and epigenetic modification.

## 2. Results

### 2.1. KDM6B Inhibited the Expression of Neural Markers in SCAPs

**Figure 1:**
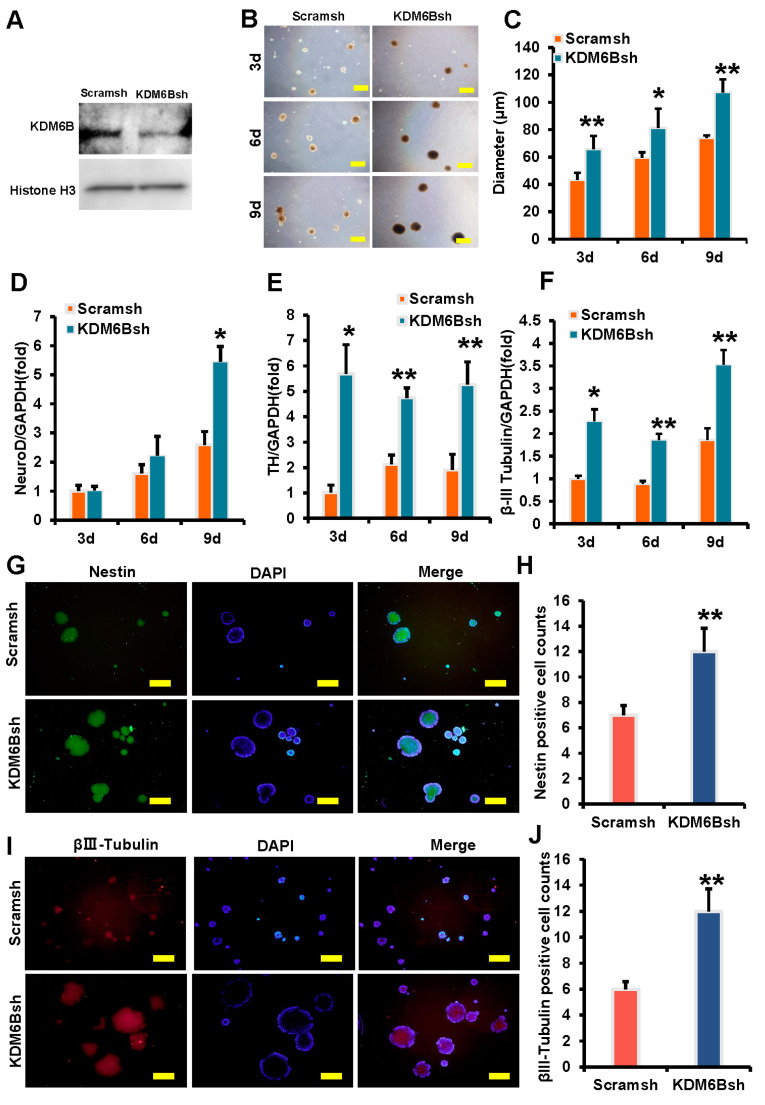
KDM6B knockdown enhanced the expression of neural markers in SCAPs. (**A**) The efficiency of KDM6B knockdown was tested by Western blot, with Histone H3 serving as an internal control; (**B**,**C**) compared with the control group, KDM6B knockdown increased the volume of neurospheres, demonstrated by a scale bar of 100 μm; (**D**–**F**) real-time RT-PCR analysis revealed that KDM6B knockdown significantly upregulated the expression of NeuroD (**D**), TH (**E**), and β-III tubulin (**F**) in SCAPs, with GAPDH as an internal control; and (**G**–**J**) immunofluorescence staining indicated that the expression levels of Nestin and β-III Tubulin, as well as the percentage of Nestin and β-III Tubulin positive cells in neurospheres, were also elevated in the KDM6Bsh group compared with the control group. Statistical significance was determined using Student’s *t*-test, and all error bars represent SD (*n* = 3). * *p* < 0.05. ** *p* ≤ 0.01.

In this study, the effect of KDM6B on neural marker expression was investigated using SCAPs. To achieve KDM6B knockdown in SCAPs, lentivirus transfection was utilized, followed by detection of knockdown efficiency through Western Blot analysis after 3 days of treatment with 2 μg/mL of puromycin. The expression level of KDM6B exhibited a significant decrease in the KDM6Bsh group when compared to the control group (Figure 1A). Morphological changes in SCAPs during neural induction were also observed, with dynamic changes recorded at 3, 6, and 9 days. The size of the neurosphere in the control group was compared with those in the KDM6Bsh group. Results showed that the average diameter of the neurosphere in Scramsh and KDM6Bsh groups were 43 and 66 μm on day 3, 59 and 81 μm on day 6, and 74 and 107 μm on day 9, respectively (Figure 1B,C). The findings showed that the neurosphere in the KDM6Bsh group exhibited larger sizes compared to those in the control group. Real-time RT-PCR analysis was used to detect the expression of neural markers, and the results showed that NeuroD was increased in the KDM6Bsh group at 3 days, and TH and β-III Tubulin were increased in the KDM6Bsh group at 3, 6, and 9 days compared with the control group (Figure 1D–F). Immunofluorescence results at 9 days indicated that the positive neurosphere of Nestin and β-III tubulin in the KDM6Bsh group exhibited significantly larger sizes, with a significant increase in the number of neurospheres recorded at 1.71 and 2 times that of the control group, respectively, with statistical significance (Figure 1G–J). These findings suggest that the knockdown of KDM6B promotes the expression of neural markers in SCAPs and enhances neurosphere formation.

**Figure 2:**
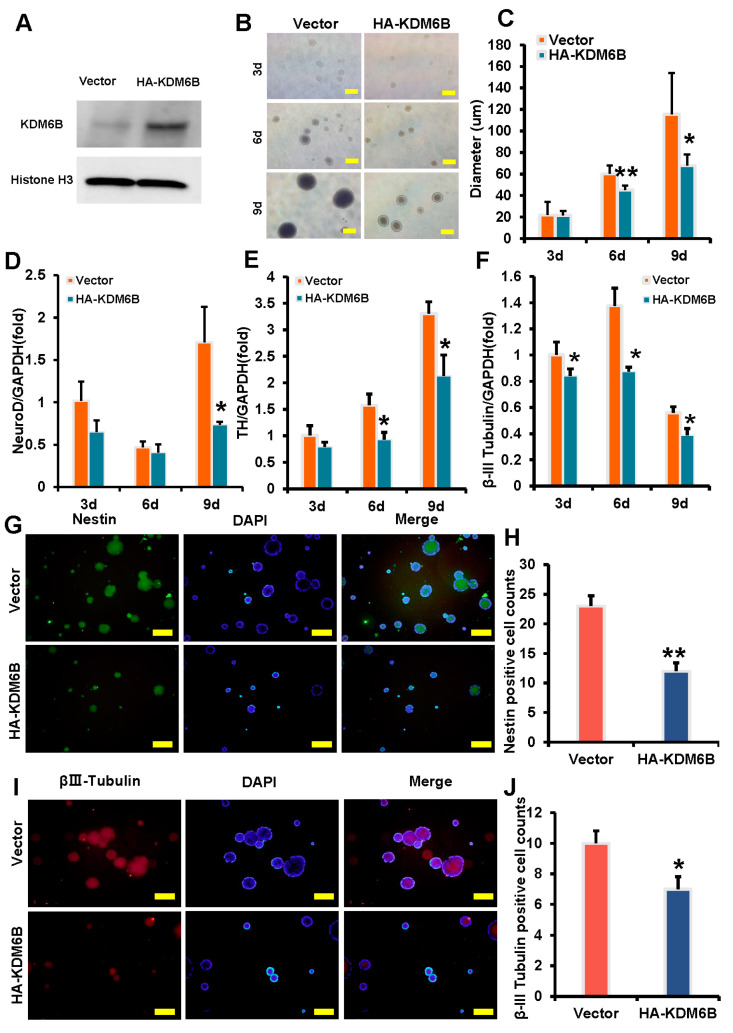
KDM6B overexpression inhibited the expression of neural markers in SCAPs. (**A**) The KDM6B overexpression efficiency was tested by Western blot, with Histone H3 serving as an internal control; (**B**,**C**) compared with the control group, KDM6B overexpression decreased the volume of neurospheres, demonstrated by a scale bar of 100 μm; (**D**–**F**) real-time RT-PCR analysis revealed that KDM6B overexpression significantly downregulated the expression of NeuroD (**D**), TH (**E**), and β-III tubulin (**F**) in SCAPs, with GAPDH as an internal control; and (**G**–**J**) immunofluorescence staining indicated that the expression levels of Nestin and β-III tubulin, as well as the percentage of Nestin and β-III tubulin positive cells in neurospheres, were also decreased in the HA-KDM6B group compared with the control group. Statistical significance was determined using Student’s *t*-test, and all error bars represent SD (*n* = 3). * *p* < 0.05. ** *p* ≤ 0.01.

To investigate the effect of KDM6B on neural marker expression in SCAPs, we overexpressed KDM6B in SCAPs and observed morphological changes during neural induction. The efficiency of KDM6B overexpression was confirmed by Western blot analysis. The expression level of KDM6B presented a marked increase in the HA-KDM6B group when compared to the control group (Figure 2A). Dynamic changes were observed at 3, 6, and 9 days, and the size of the HA-KDM6B neurosphere was compared to those of the control group. The average diameter of the neurosphere in the control and HA-KDM6B groups was measured at different time points, with statistically significant differences between the groups. The results showed that compared with the control group, the volume of neurospheres in the HA-KDM6B group was smaller (Figure 2B,C). Real-time RT-PCR analysis revealed that neural markers such as NeuroD at 3 days, TH at 6 and 9 days, and β-III tubulin at 3, 6, and 9 days were downregulated in SCAPs following KDM6B overexpression compared to the control group (Figure 2D–F). Immunofluorescence analysis on day 9 showed that the positive neurosphere of Nestin and β-III tubulin in the HA-KDM6B group were significantly smaller than those in the control group, with a reduction in the number of positive cells, 47.8% and 30%, respectively (Figure 2G–J).

### 2.2. KDM6B Knockdown Significantly Promoted SCAP-Mediated Locomotor Recovery after Complete Transection of the Rat Spinal Cord

**Figure 3:**
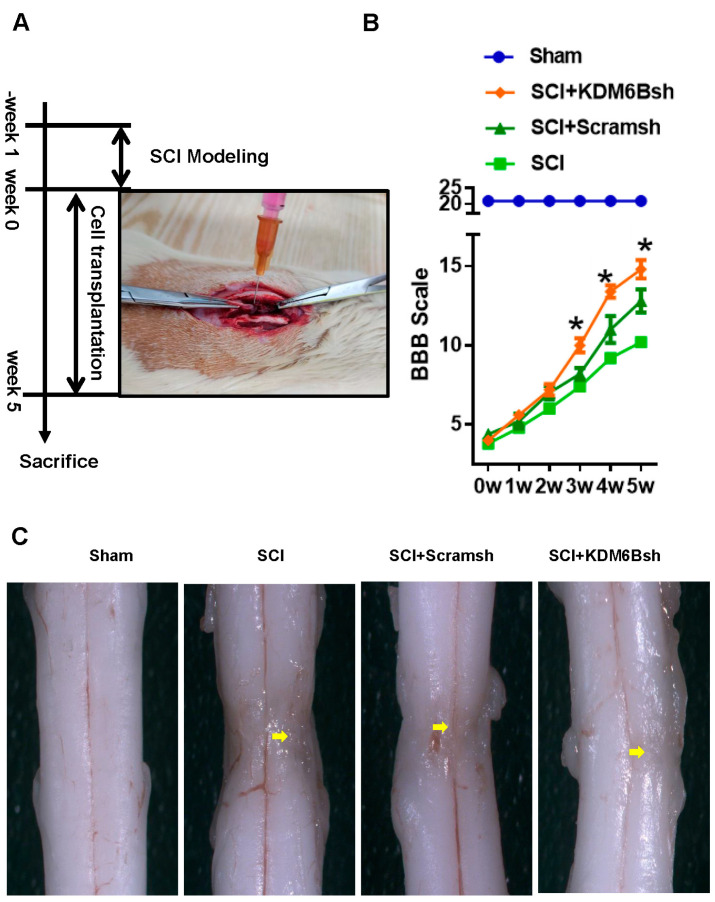
The morphological and functional changes after SCAP transplantation in SCI model. (**A**) The limb motor function of rats was assessed using the BBB scale at 1–5 weeks post-SCI; (**B**) compared with the Scramsh group, the KDM6Bsh group exhibited a gradual improvement in spinal function recovery from week 3 to 5, as demonstrated by the BBB scale; and (**C**) the spinal cord morphology and histological structures of the KDM6Bsh group were found to be better recovered compared with those of the SCI and Scramsh groups. The yellow arrows depict regions of spinal cord injury. Statistical significance was determined using the one-way ANOVA and Kruskal–Wallis test and all error bars represent SD (*n* = 6). * *p* < 0.05.

We established a spinal cord hemisection rat model and transplanted SCAPs to evaluate their repair effect after 5 weeks (Figure 3A). The BBB scale was used to assess functional recovery. Results showed that the BBB scale of the three groups fluctuated at around 5 during weeks 0–2, with no statistical differences among these three groups. However, in week 3, the KDM6Bsh group had a higher scale (9.83 ± 0.98) than the Scramsh group (8.33 ± 0.82) and the SCI group (7.67 ± 1.03). In week 4, the KDM6Bsh group scaled 12.83 ± 0.98 while the Scramsh group and the SCI group scaled 11.17 ± 1.72 and 9.33 ± 0.82, respectively. In the fifth week, the scales further increased to 10.50 ± 1.38, 12.67 ± 1.51, and 15.00 ± 1.27 in the SCI group, Scramsh group, and KDM6Bsh group, respectively (Figure 3B). The scales of each group increased during weeks 1–5, but the SCI + KDM6Bsh group demonstrated higher scales than the other two groups, with statistically significant differences. A total of 5 weeks after transplantation, examination under a microscope revealed obvious tissue depression, scar formation, uneven surface, and color variation in the area of SCI mold construction in the SCI model group, indicating typical inflammatory repair. However, the SCI + Scramsh group showed some improvement in spinal tissue, with more plump white tissue appearing and reduced repair tissue traces. The SCI + KDM6Bsh group displayed satisfactory tissue repair, with almost no trace of tissue shrinkage in the modeling area, good fusion with surrounding tissues, and normal spinal cord morphology (Figure 3C).

**Figure 4:**
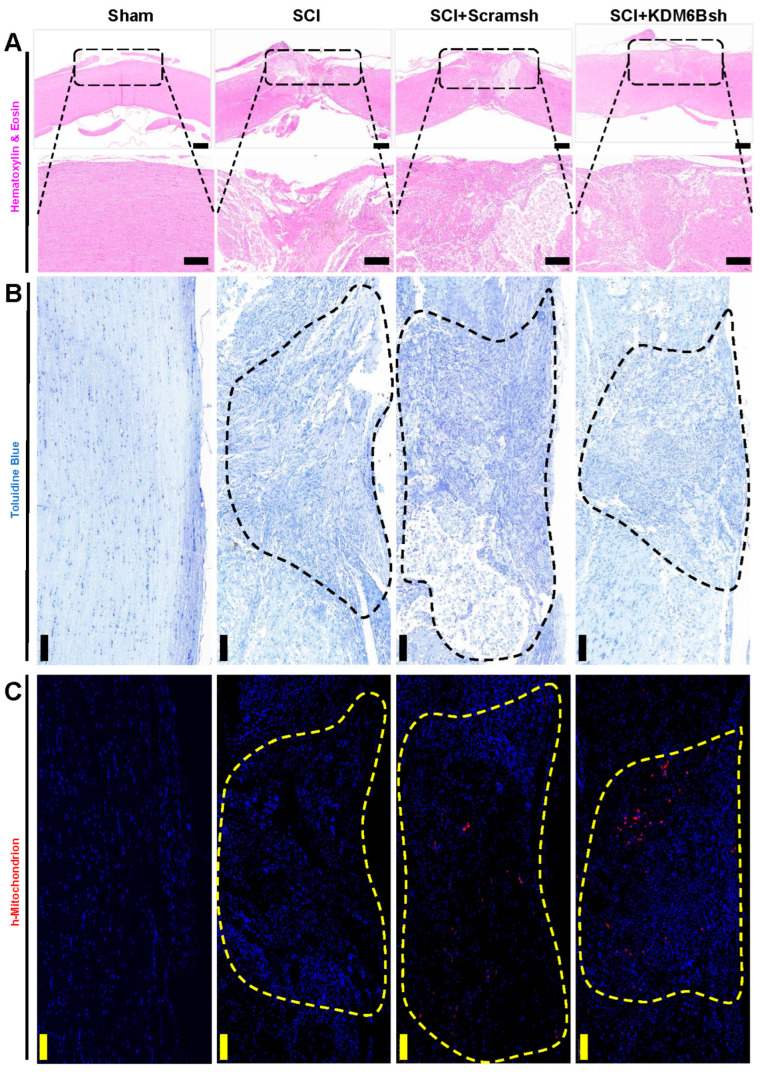
The pathological changes after SCAP transplantation in SCI model. (**A**) HE staining result revealed that the KDM6Bsh group had fewer cavities and scars compared with the SCI and Scramsh group. Scale bar, 100 μm, and 500 μm; (**B**) toluidine blue staining result revealed that obvious tissue cavities were observed in the SCI group, and the KDM6Bsh group showed very fine cavities compared with the Scramsh group; and (**C**) the immunofluorescence results indicated that h-mitochondria-positive cells were in the injury area of Scramsh and KDM6Bsh groups. Scale bar, 50 μm.

To further assess the repair of spinal cord tissue, we stained the repair tissue using hematoxylin–eosin (HE) and toluidine blue (TB). HE staining was employed to visualize tissue cell structure, while TB staining was used to assess the degree of neuronal damage repair. HE staining showed that there were large tissue vacancies in the SCI group, while the SCI + Scramsh group displayed only a few tissue cavities resembling residual scar tissue. However, the tissue cavities in the SCI + KDM6Bsh group were almost invisible, closely resembling the spinal cord tissue of the sham group (Figure 4A). Results from TB staining of the neural matrix were consistent with those from HE staining. In the SCI group, obvious tissue cavities were observed in the defect area, and the SCI + Scramsh group exhibited significant non-colored tissue. In contrast, the SCI + KDM6Bsh group only showed very fine cavities, similar to the staining of spinal cord tissue in the sham group (Figure 4B). To assess the distribution of SCAPs, we utilized h-Mitochondrion staining. H- Mitochondrion can serve as a reliable marker for identifying transplanted SCAPs. Specifically, the distinctive properties of mitochondrial structure and function allowed us to accurately identify the location of the transplanted SCAPs within the host organism. Fluorescence results revealed the absence of any fluorescence signal in both the sham operation group and the SCI group. However, scattered h-mitochondrion staining was observed in the SCI + Scramsh and SCI + KDM6Bsh groups, indicating the presence of transplanted SCAPs within the tissue of the defective region (Figure 4C).

**Figure 5:**
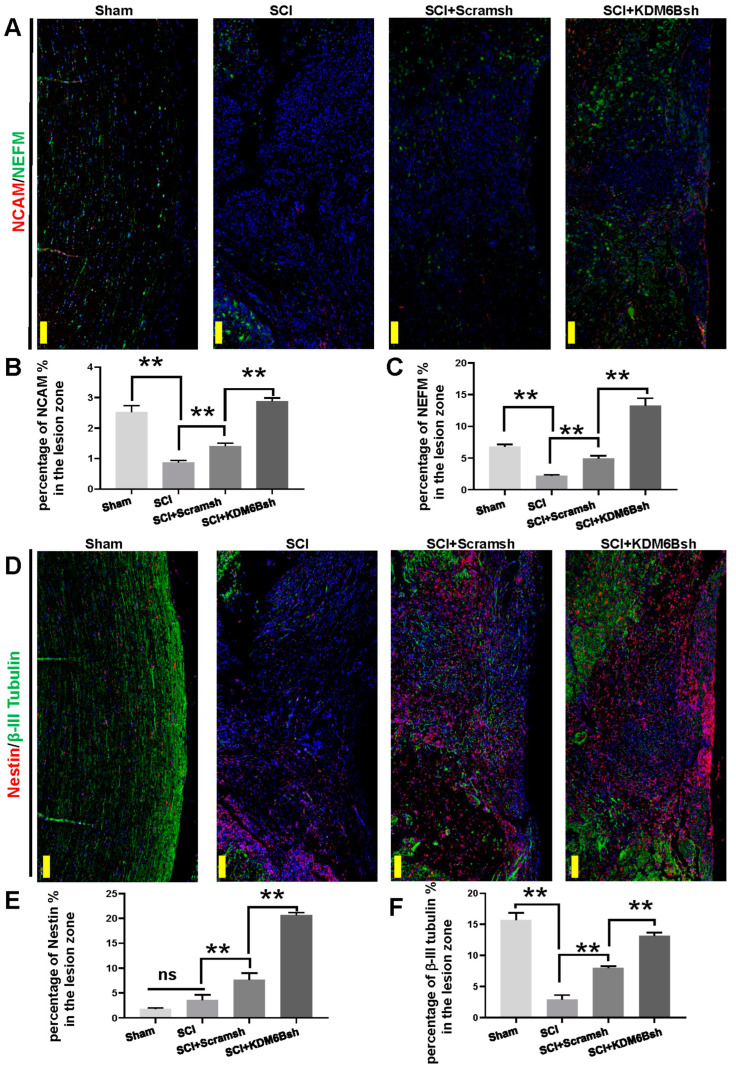
The neural marker expression after SCAP transplantation in SCI model. (**A**) The immunofluorescence staining of NCAM and NEFM. Scale bar, 500 μm; (**B**) statistical analysis of the percentage of NCAM in the lesion zone in four groups; (**C**) statistical analysis of the percentage of NEFM in the lesion zone in four groups; (**D**) the immunofluorescence staining of Nestin and β-III tubulin. Scale bar, 500 μm; (**E**) statistical analysis of the percentage of Nestin in the lesion zone in four groups; and (**F**) statistical analysis of the percentage of β-III tubulin in the lesion zone in four groups. Statistical significance was determined using the one-way ANOVA and Kruskal–Wallis test. ns: no significant difference. ** *p* ≤ 0.01.

To examine neuronal communication and intracellular filament distribution, we stained the NCAM (Neural Cell Adhesion Molecule) and NEFM (Neurofilament Medium), respectively (Figure 5A). The expression of NEFM and NCAM may serve as reliable indicators of spinal cord injury repair. The results of NCAM immunofluorescence analysis indicated a significant decrease in the expression of NCAM in the SCI group compared to the Sham group. The KDM6Bsh group exhibited a significant increase in NCAM expression when compared to both the SCI and Scramsh groups (Figure 5A,B). Similar results were observed for NEFM immunofluorescence analysis, where the expression of NEFM was lower in the SCI group than in the Sham group. However, the KDM6Bsh group displayed a higher level of NEFM expression in the spinal cord defect compared to both the SCI and Scramsh groups (Figure 5A,C). The localization of neural progenitor cells in spinal cord defects was evaluated using fluorescence staining of Nestin (Figure 5D). A small amount of Nestin fluorescence signal was observed in the SCI group, with no significant difference noted between the SCI and Sham groups. The Scramsh group exhibited a higher expression of Nestin than the SCI group, yet it remained significantly lower than that observed in the KDM6Bsh group (Figure 5D,E). The fluorescence staining of β-III Tubulin was used to assess nerve repair performance by displaying the elongation behavior of axons. Results showed a significant decrease in the expression of β-III Tubulin in the SCI group compared to the Sham group. While the expression of β-III Tubulin in the Scramsh group was higher than that in the SCI group, it still remained lower than that observed in the KDM6Bsh group (Figure 5D,F).

### 2.3. Differentially Expressed Genes and Bioinformatic Analysis in KDM6B Overexpressed SCAPs

To identify differentially expressed genes between KDM6B-overexpressed SCAPs and the control group, we performed microarray analysis. The results indicated 118 differential genes in the KDM6B overexpressed SCAPs compared to the control group. Among these genes, 40 were upregulated, while 78 were downregulated (Supplementary Table S2). Specifically, upregulated genes included MMP1, FAM111B, RPS27A, MAF, FOXG1, and COL14A1, while downregulated genes included NR4A1, N4RA2, HES1, EGR1, PTGS2, FOSB, and FOS.

**Figure 6:**
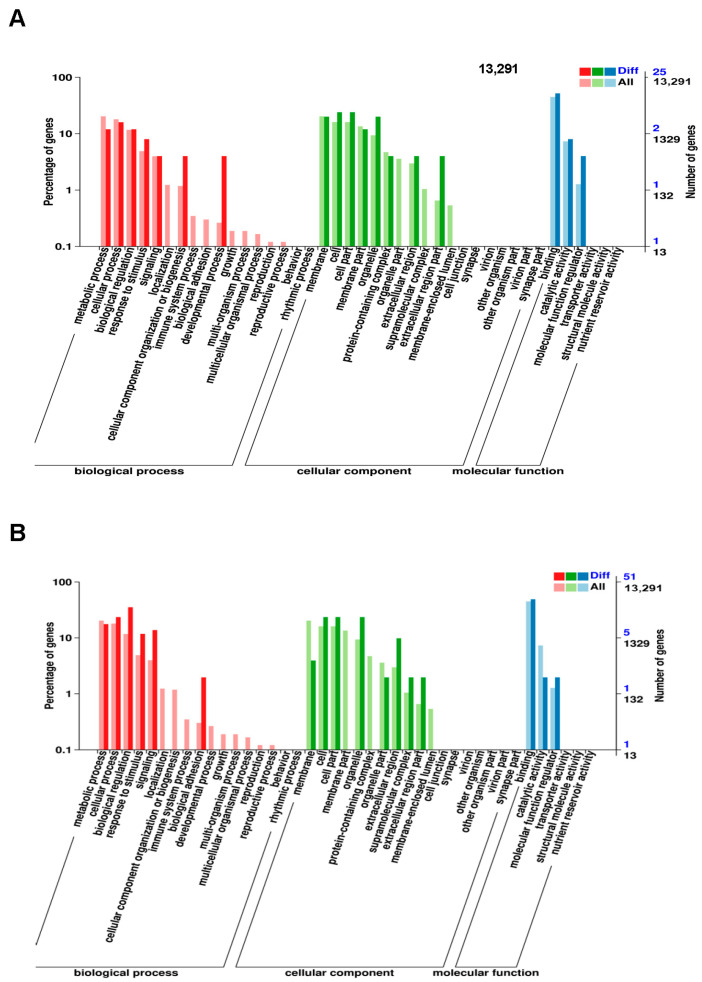
Significant gene ontology (GO) analyses of differentially expressed genes in KDM6B overexpressed SCAPs compared with the control group. The GO functions were represented by the *x*-axis, while the *y*-axis indicated the percentage and number of genes associated with each function. The results revealed that there were distinct GO functions associated with upregulated (**A**) and downregulated genes (**B**).

**Figure 7:**
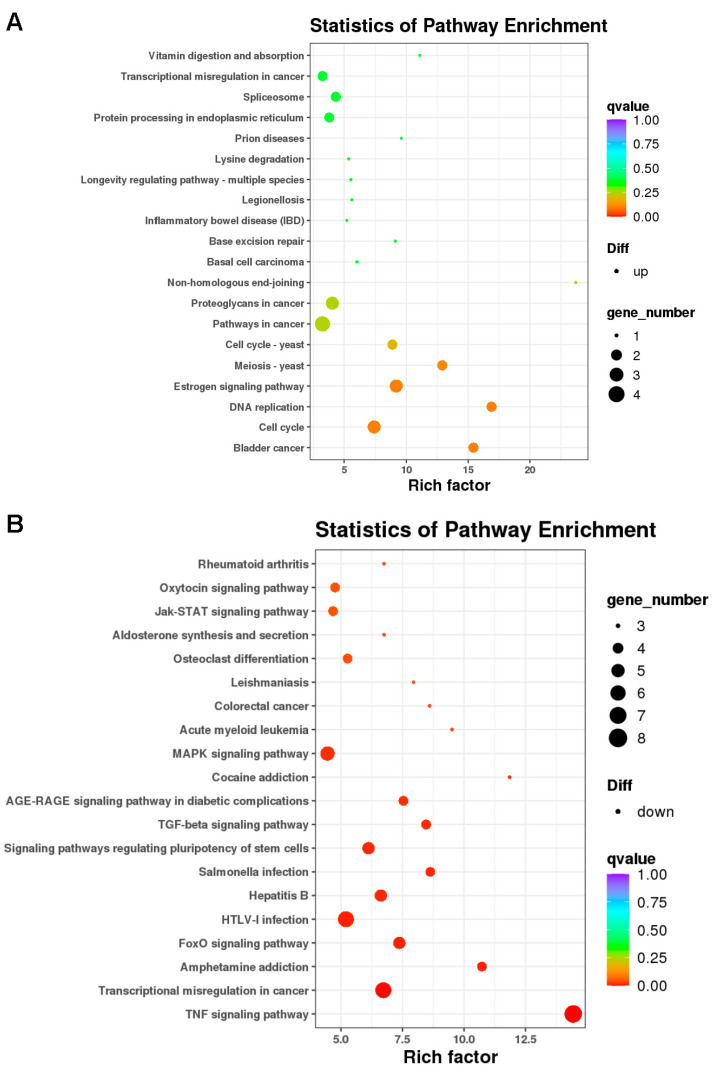
Statistics of KEGG pathway enrichment of differentially expressed genes in KDM6B overexpressed SCAPs compared with the control group. The statistics of KEGG pathway enrichment of upregulated genes (**A**) and downregulated genes (**B**) during KDM6B overexpression. The statistical analysis is presented in circular plots, where each circle represents a KEGG pathway. The number of annotated genes for different KEGG terms is indicated by circular dots, while different colors represent various levels of significance (*p*-value). The rich factor, which reflected the ratio of gene numbers for each term to the numbers of all genes with terms, is also provided.

We carried out gene ontology (GO) analysis and pathway analysis to identify the statistical enrichment of various biological functions and pathways associated with these differentially expressed genes. GO analysis showed that upregulated functions included catalytic activity, binding, cellular processes, and biological regulation, while downregulated functions included negative regulation of the metabolic process, cellular processes, signaling, and biological regulation (Figure 6A,B). Pathway analysis using the KEGG database identified significant alterations in differentially expressed genes and highlighted pathways involved in KDM6B function regulation. The enrichment degree was based on the *p*-value and enrichment factor. Specifically, upregulated genes were associated with the estrogen signaling pathway, DNA replication, and cell cycle, while downregulated genes were associated with the Jak-STAT signaling pathway, MAPK signaling pathway, TGF-beta signaling pathway, TNF signaling pathway, signaling pathways regulating the pluripotency of stem cells, and FoxO signaling pathway (Supplementary Table S3 and Figure 7A,B).

### 2.4. KDM6B Negatively Regulated the HES1 by Binding to the HES1 Promoter in SCAPs

**Figure 8:**
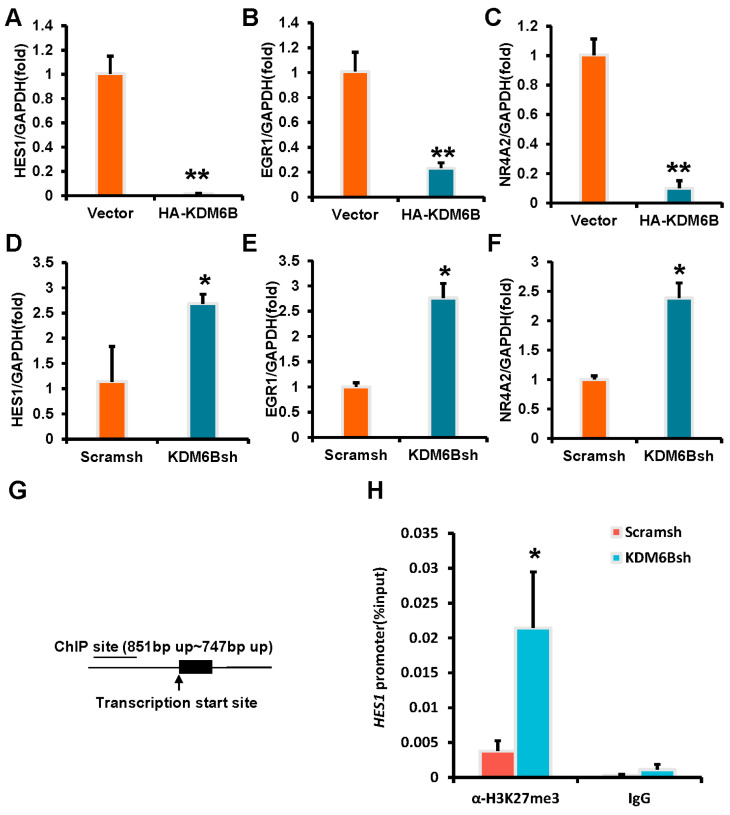
Knockdown of KDM6B directly increases the H3K27me3 enrichment in the HES1 promoter. (**A**–**C**) Real-time RT-PCR analysis was conducted on the HA-KDM6B group, which showed a significant reduction in the expression of HES1 (**A**), EGR1 (**B**), and NR4A2 (**C**). (**D**–**F**) Real-time RT-PCR analysis was performed on SCAPs, revealing an upregulation of HES1 (**D**), EGR1 (**E**), and NR4A2 (**F**) expression. GAPDH was used as an internal control. (**G**,**H**) ChIP experiment results revealed that KDM6B depletion increased the enrichment of H3K27me3 in the HES1 promoter. Statistical significance was determined using Student’s *t*-test, with all error bars representing SD (*n* = 3). * *p* < 0.05 and ** *p* ≤ 0.01.

Three downregulated genes, HES1, EGR1, and NR4A2, were selected from the microarray analysis and used real-time RT-PCR to detect their expression after KDM6B knockdown and overexpression. The results were consistent with the microarray analysis data, and in SCAPs overexpressing KDM6B, the expressions of HES1, EGR1, and NR4A2 were significantly downregulated (Figure 8A–C), and the knockdown of KDM6B upregulated the expressions of HES1, EGR1, and NR4A2 (Figure 8D–F). To verify that these three genes are direct downstream targets of KDM6B action, we performed ChIP (Chromatin Immunoprecipitation) experiments in KDM6B knockdown SCAPs. The ChIP results showed that the H3K27me3 element was significantly enriched in the HES1 promoter in the KDM6Bsh group compared to the control group, indicating that KDM6B may directly regulate the expression of HES1 through H3K27me3 (Figure 8G,H). However, no statistically significant difference was found in the EGR1 and NR4A2 promoters in the KDM6Bsh group and the control group (data are not shown).

## 3. Discussion

Nerve injury caused by trauma and tumors seriously affects patients’ quality of life. Current treatments mainly rely on surgical decompression, neurotrophic drugs, and physical therapy. However, their efficacy cannot meet clinical needs [24,25]. In recent years, stem cell therapy has emerged as a promising strategy for repairing nerve tissue. Stem cells secrete trophic factors, regulate inflammation, induce axon extension, and inhibit scarring [5,6,7,8]. SCAPs, a type of mesenchymal stem cell, have distinct advantages in terms of acquisition, stemness, and neuro-nutrition [21]. Nevertheless, the underlying mechanism by which SCAPs derived from mesoderm promotes the repair and regeneration of ectodermal differentiated neural tissue remains unclear. Epigenetic regulatory mechanisms such as histone methylation have only recently been discovered by generating function-specific neurons or mimicking neurological diseases. However, the role of histone methylation or demethylation in human neurogenesis and related diseases, particularly during neuroectodermal succession, is still poorly understood. KDM6B, an enzyme that modifies histones, plays an essential role in early neurogenesis. Inhibition of its expression can promote neuroectodermal differentiation of human pluripotent stem cells [19]. In this study, we aim to analyze the biological role and mechanism of KDM6B in repairing neural tissue using SCAPs.

The present study investigated the role of KDM6B in the development of neuron-like characteristics in SCAPs. The knockdown of KDM6B led to the formation of larger neuron-like cells, which displayed a typical morphological manifestation of neurogenesis induction [26]. Immunofluorescence staining revealed that the depletion of KDM6B has a significant increase in the number of positive cells for Nestin and β-III Tubulin. Nestin is an intermediate filament protein that is expressed in neural precursor cells, and it is involved in maintaining the proliferation capacity of these cells. β-III Tubulin is a microtubule protein that is highly expressed in mature neurons. Its expression is also upregulated in neural progenitor cells during neurogenesis. The expression of Nestin and β-III Tubulin serves as a useful indicator of the potential for neural regeneration after a spinal cord injury [27,28]. The upregulation of the expression of Neurod, TH, and β-III Tubulin was also observed after KDM6B knockdown, suggesting that SCAPs were inclined to differentiate into neuron-like cells during induction. Neurod is an essential factor for maintaining the maturity and survival of neurons and is highly expressed in neurons [29], while TH plays a crucial role in the synthesis and secretion of dopamine, a vital neurotransmitter [30]. Moreover, the upregulation of β-III Tubulin expression in perikarya and neurites of astrocytes and neurons indicates active cell structure shaping in neural tissues [31,32]. In contrast, KDM6B overexpressed SCAPs exhibited the opposite trend. Taken together, these findings suggest that KDM6B regulates the molecular expression of neuron-like cells, nerve cell structure, and neurotransmitter synthesis and secretion, making it a crucial regulatory target in the process of nerve repair.

Animal experiments were carried out to confirm the effectiveness of KDM6B knockdown on nerve tissue regeneration. The transplantation of KDM6B-knockdown SCAPs led to faster and better motor function recovery after 5 weeks, as indicated by BBB scores, reaching about 70% of the sham group. Morphologically, the spinal tissue at the defect of the KDM6B depleted group exhibited satisfactory results in terms of texture, morphology, and fusion with the surrounding area. HE staining and toluidine blue staining revealed that the distribution of cells and stroma in the defective area of the spinal cord treated with KDM6B-knockdown SCAPs was similar to that in the normal spinal cord, indicating a better repair effect.

Moreover, the observation of h-mitochondria revealed that transplanted human SCAPs remained localized within the operative area of spinal cord defects. It can be concluded that SCAP transplantation promotes the activation of neural precursor cells, the remodeling of neuronal axons, and the information exchange between nerve cells in the spinal cord injury model of rats, forming favorable conditions for nerve tissue regeneration and signal transduction, with KDM6B knockdown further enhancing this effect. NCAM is involved in axon growth and guidance, synaptic function, and neuronal survival [33,34]. Its expression is upregulated after a spinal cord injury, indicating the activation of neuronal repair mechanisms. Our study found that NCAM content in the KDM6B knockdown group was higher in the new tissue of the defective nerve, which represents enhanced cell–cell interaction at the site of the defective tissue. NEFM is a protein that is specifically expressed in large myelinated axons, and its presence indicates the potential for remyelination and restoration of normal axonal function [35,36]. However, abnormal changes in NEFM expression are often indicative of neural tissue injury [37]. In our study, we found that the KDM6B knockdown group expressed a higher level of NEFM. The disorder of we suspect that this phenomenon could facilitate the development of neural connectivity and the transfer of signals within the nervous system. Additionally, Nestin and β-III tubulin, two cytoskeletal proteins located in neural precursor cells and neurons, respectively, were regarded as specific molecular markers [38]. SCAP transplantation after spinal cord injury modeling enhanced the expression of Nestin and β-III tubulin, suggesting that SCAPs may regulate the microenvironment in the nerve defect area where neural stem cells are located and are beneficial to the reconstruction of neural tissue.

To investigate the effects of KDM6B on SCAP regulation in the neural repair microenvironment, we conducted a microarray analysis to identify differentially expressed genes and signaling pathways. The expression levels of hairy and enhancer of split 1 (HES1), EGR1 (early growth response 1), and nuclear receptor subfamily 4 (NR4A2) were significantly downregulated in KDM6B overexpressed SCAPs. HES1 is a direct transcriptional target of the Notch pathway and plays a critical role in determining the fate of neural precursor cells [39]. NOTCH signal contributes to the stable transformation of neural precursor cells into neurons [40,41]. EGR1 is involved in various neuronal processes, including neurotransmission, synaptic plasticity, and advanced behaviors such as learning, memory, emotion, and reward [42]. NR4A2 is a nuclear receptor and transcription factor that regulates the differentiation, survival, and protection of dopaminergic neurons [43]. Furthermore, the ChIP assay showed that H3K27me3 was enriched into the HES1 promoter region and regulated its expression in KDM6B knockdown SCAPs. These results showed that HES1 was the downstream gene regulated by KDM6B. Despite the progress made in this study, there are still opportunities for further development. It is widely recognized that numerous genes and pathways are involved in neurodevelopment. While HES1 acts as a key regulator of the Notch pathway, the microarray analysis conducted in this research highlights more significant expression differences in the JAK-STAT signaling pathway, estrogen signaling pathway, and MAPK pathway. Further investigation is necessary to elucidate the connections and differences between these mechanisms. Moreover, previous studies have predominantly focused on neural stem and progenitor cells, which can differentiate into neurons and other cell types. In contrast, this study primarily used SCAPs to stimulate the secretion of nerve-related growth factors. The question of whether ectopic tissue-derived stem cells, such as SCAPs, can be induced to differentiate into neurons and play a therapeutic role in clinical settings remains an important area for future investigation.

The findings of this research have significant implications for the treatment of spinal cord injury. Currently, there are limited effective therapies available to promote functional recovery after spinal cord injury. The identification of KDM6B as a potential therapeutic target for improving motor dysfunction post-SCI is a meaningful discovery in the field. Additionally, our study demonstrates that combining KDM6B treatment with SCAP transplantation can provide a more effective strategy for promoting recovery after spinal cord injury. This combination approach has the potential to significantly improve the quality of life for individuals suffering from spinal cord injury.

## 4. Materials and Methods

### 4.1. Cell Cultures

SCAPs used in this study were procured from Shanghai Anwei Biotechnology Co., Ltd. (Shanghai, China). SCAPs were extracted from the immature root tip tissue of wisdom teeth with appropriate patient consent. SCAP cultures at the third to fifth passages were utilized for conducting the subsequent experiments. Further details regarding the culture of SCAPs have been elucidated in our previous publication [44].

### 4.2. Plasmid Construction and Viral Infection

In this study, plasmids were utilized as per standardized protocols. The pQCXIN retroviral vector was used for the insertion of the hemagglutinin (HA)-tag, along with the complete sequence of KDM6B, while the pLKO.1 lentiviral vector was employed for the insertion of short hairpin RNA (shRNA) specific to KDM6B. The virus packaging procedure was performed meticulously in accordance with the guidelines provided by Clontech Laboratories or Addgene, depending on the specific protocol adopted. The shRNA target sequence for KDM6B was reported as KDM6B shRNA (KDM6Bsh) 5′-AAGTGGAATGTTTAACAGTGC-3′, and the scramble shRNA (Scramsh) was procured from Addgene.

### 4.3. Neurogenic Differentiation and Immunofluorescence Staining

For the induction of neurogenic differentiation in SCAPs, we seeded 1×10^6^ cells into ultra-low adsorption dishes (Coring, Houston, TX, USA) and cultured them for 9 days. The neural induction medium employed was previously described [45]. Half changes of neural induction medium were carried out every 3 days. The progress of neuron-like cell differentiation was monitored under a microscope at 3 days, 6 days, and 9 days after neural induction. During the neural induction process, three to five images were captured at ×10 magnification using an Olympus IX71 microscope on days 3, 6, and 9. The diameter of the resulting neural spheres was measured post-image acquisition using Image J software. After completing the 9-day induction process, the neurospheres were harvested and subjected to centrifugation at 1100 rpm for 5 min. Subsequently, the microspheres were re-suspended in 100 μL of nerve induction medium, and all cells were quantified via immunofluorescence analysis. Immunofluorescence staining was performed to detect the markers of neurogenesis, as described in our previous publication [25]. At 6 weeks post-surgery, rats from all experimental groups were anesthetized with 1% sodium phenobarbital (4 mL/kg) and transcardially perfused with 0.9% saline, followed by fixation using 4% paraformaldehyde. Subsequently, 10 μm thick sections were prepared and mounted onto slides for immunofluorescent staining analysis. The immunofluorescence staining protocol was carried out as detailed previously [46,47].

The primer antibodies were as follows: Nestin (1:100, Cat No. ab 6320, Abcam, Cambridge, UK), β-III-Tubulin (1:400, Cat No. ab68193, Abcam, Cambridge, UK), NEFM (1:100, Cat No. A16405, ABclonal, Wuhan, China), NCAM (1:100, Cat No. ab75813, Abcam, Cambridge, UK), and h-mitochondria (1:100, Cat No. ab 92824, Abcam, Cambridge, UK).

### 4.4. Western Blot Analysis

Protein extraction and gel electrophoresis methods employed in this study were consistent with the protocols outlined in our previous publication [48]. The primary antibodies used for detection included KDM6B (1:400, Cat No. 07-1533, Millipore, Temecula, CA, USA) and Histone H3 (1:1000, Cat No. SC-56616, Santa Cruz, CA, USA).

### 4.5. Real-Time Reverse Transcriptase-Polymerase Chain Reaction (Real-Time RT-PCR)

Total RNA was extracted from SCAPs using an extraction kit (RC112-01, Vazyme, Nanjing, China). The extracted RNA (1 μg) was reverse transcribed into cDNA according to the standard procedure of the Invitrogen kit. Real-time PCR reactions were carried out in a manner consistent with our previous publication [49]. Glyceraldehyde 3-phosphate dehydrogenase (GAPDH) served as an internal reference gene for analysis of relative mRNA levels. The primers employed in this study are listed in Supplementary Table S1.

### 4.6. Spinal Cord Injury Model

Female Sprague Dawley rats aged 6–8 weeks were procured from Vital River Laboratory Animal Technology Co., Ltd. (Beijing, China) for use in the spinal cord injury (SCI) model. Following a one-week period of adaptive feeding, surgical procedures were performed on all rats. Anesthesia was induced in the rats using sodium pentobarbital (40 mg/kg, ip), following which the spine between T9 and T10 was exposed, and hemisection of the spinal cord at T10 was performed as per previously established methods [47]. A suspension containing 2 × 10^6^ cells in 5 μL volume was added to a 30 μL ECM gel (Cat No. E1270, Sigma, St. Louis, MO, USA) and transplanted at the site of spinal cord injury. The Sprague Dawley rats were randomly assigned to four groups: Sham, SCI, SCI + Scramsh, and SCI + KDM6Bsh, (*n* = 6).

### 4.7. Behavioral Testing

To assess the exercise ability of the rats, we employed the Basso, Beattie, and Bresnahan locomotor rating scale (BBB scale) [50]. Two experimenters who were blinded to the treatment group evaluated each rat. The evaluation process involved assessing limb movement, gait, coordination, and paw placement at 1 week, 2 weeks, 3 weeks, 4 weeks, and 5 weeks after injury.

### 4.8. Histological Analyses and Toluidine Blue Staining

At 5 weeks post-SCAPs transplantation, the rats were sacrificed, and spinal cords containing the injured site were collected. The collected spinal cord tissue was fixed for 24 h using 4% paraformaldehyde and 30% sucrose solution at 4 °C. Tissue sections of 5 μm thicknesses were prepared and stained with hematoxylin–eosin (HE). To evaluate the number and degranulation of mast cells, the spinal cord sections were subjected to toluidine blue staining following standard protocols. Initially, the sections were deparaffinized in xylene and then dehydrated through a series of graded ethanol solutions for 5 min each (Cat No. B1007, Wuhan Bioqiandu Technology, Wuhan, China). Subsequently, they were placed in water for 5 min and transferred to toluidine blue for 4 min before being carefully blotted. Following this, the sections were treated with absolute alcohol for 1 min, cleared in xylene, and mounted on a glass slide using Eukitt (Bio-Optica, Milan, Italy). The staining protocol was consistent with established procedures and resulted in blue-stained sections and purple-stained mast cells [51].

### 4.9. Microarray Analysis

For gene expression profiling, the PrimeView™ Human Gene Expression Array was employed. The differential gene expression analysis was carried out using Affymetrix GeneChip Operating Software, as per previously established methods [52]. Differentially expressed genes were selected based on a fold change > 1.5 and a *p*-value < 0.05 as the threshold criteria for identifying significant changes in mRNA expression.

### 4.10. Chromatin Immunoprecipitation (ChIP) Assays

The ChIP assay was conducted utilizing a ChIP assay detection kit (Merck Millipore, Darmstadt, Germany), following established protocols [49]. The DNA precipitation step was carried out using anti-H3K27me3 antibody (Cat No. 9733s, Cell Signaling Technology, Danvers, MA, USA). The quantification of the ChIP assay results was expressed as a percentage of input DNA. The primers used for targeting the promoter region of the target gene were HES1 promoter binding site (HES1-BS), with forward primer sequence 5′-ACCCTGGCTCCAAAAGAAAT-3′ and reverse primer sequence 5′-TACTCTCCCTCTGGGCTTTG-3′.

### 4.11. Statistical Analyses

Each experiment was conducted a minimum of 3 times to ensure the reliability of the results. Statistical calculations were carried out using GraphPad5 (GraphPad Software, La Jolla, CA, USA) or SPSS 17 (IBM Corporation, New York, NY, USA). The choice of statistical test was determined based on the nature of the dataset and its distribution, with either Student *t*-test, one-way ANOVA, or Kruskal–Wallis test employed accordingly. A *p*-value ≤ 0.05 was considered statistically significant for all analyses.

## 5. Conclusions

This study aimed to investigate the role of KDM6B in promoting SCAP-mediated nerve repair. We observed that SCAPs could enhance adhesion and spreading between neurons, while knockdown of KDM6B led to increased expression of neural markers and secretion of the neural matrix. Furthermore, we identified the KDM6B-HES1 signaling axis as a key mechanism involved in this process. Overall, these findings shed new light on the potential for epigenetic modification to assist SCAPs in promoting nerve repair, and provide a basis for future studies for dental tissue-derived stem cells in treating nerve defects.

## Data Availability

Not applicable.

## Supplementary Materials

The following supporting information can be downloaded at https://www.mdpi.com/article/10.3390/ijms241310608/s1.
