# Supplementary material for: KDM6B Negatively Regulates the Neurogenesis Potential of Apical Papilla Stem Cells via HES1
Source: Int J Mol Sci. 2023 Jun 25;24(13):10608. doi: 10.3390/ijms241310608 (PMC10341966; doi:10.3390/ijms241310608)
Supplement: Supplementary file 1 [file ijms-24-10608-s001.zip › ijms-2428922-supplementary/Supplementary Table S1.pdf]

Supplementary Table S1. Primers sequences used in the Real-time RT-PCR

Gene Symbol	Primer Sequences (5'-3')
GAPDH-F	CGGACCAATACGACCAAATCCG
GAPDH-R	AGCCACATCGCTCAGACACC
NeuroD-F	CGACTGACCCCTACTCCTACCAGTCG
NeuroD-R	TGGAAGACATGGGAGCTGTCC
TH-F	CCGAGCTGTGAAGGTGTTTGA
TH-R	CGGGCCGGGTCTCTAGAT
βIII-Tubulin-F	GGCCAAGGGTCACTACACG
βIII-Tubulin-R	GCAGTCGCAGTTTTCACACTC
HES1-F	CCACCCCTCCTCCTAAACTC
HES1-R	TCCTCTTCTCTCCCAGTATTCA
NR4A2-F	TGTTGGGATGGTCAAAGAAG
NR4A2-R	TTGGACCTGTATGCTAATCG
EGR1-F	CCACCACGTACTCCTCTGTT
EGR1-R	GGTTGCTGTCATGTCCGAAA
FOSB-F	GGATCCAACCGGGCTGCAAGATC
FOSB-R	GTCGACAAAGAGTTCACCGAGC
FOS-F	TTACTACCACTCACCCGCAG
FOS-R	AGTGACCGTGGGAATGAAGT
ATF3-F	GGTTAGGACTCTCCACTCAA
ATF3-R	AGACAGTAGCCAGCGTCCTT
