# Supplementary material for: KDM6B Negatively Regulates the Neurogenesis Potential of Apical Papilla Stem Cells via HES1
Source: Int J Mol Sci. 2023 Jun 25;24(13):10608. doi: 10.3390/ijms241310608 (PMC10341966; doi:10.3390/ijms241310608)
Supplement: Supplementary file 1 [file ijms-24-10608-s001.zip › ijms-2428922-supplementary/Supplementary Table S2.pdf]

Supplementary Table S2. The differentially expressed genes in KDM6B overexpressed SCAP compared with the Vector Group

Gene Symbol	mRNA_Accession	Fold Change	p-value	diffState
NR4A1	NM_001202233; NM_001202234; NM_002135; NM_173157; NM_173158; XM_005268822; XM_005268824; XM_006719363; XM_006719364; XM_011538250; XM_011538251	-21.63	0.000000252	down
NR4A2	NM_006186; NM_173171; NM_173172; NM_173173; XM_005246621; XM_005246622; XM_005246623; XM_006712553; XM_011511246; XR_427087	-8.82	0.000013	down
HES1	NM_005524	-7.86	0.000003	down
EGR1	NM_001964	-7.16	0.000003	down
FOSB	NM_001114171; NM_006732; XM_005258691	-6.7	0.000000963	down
FOS	NM_005252	-6.52	0.000004	down
NR4A3	NM_006981; NM_173198; NM_173199; NM_173200; XM_005252237; XM_011519048; XM_011519049	-4.1	0.000019	down
FAM71A	NM_153606	-3.96	0.000054	down
IL6	NM_000600; XM_005249745; XM_011515390; XM_011515391	-3.94	0.000003	down
PTGS2	NM_000963	-3.83	0.000014	down
ID4	NM_001546	-3.82	0.000118	down

LOC102724428	NM_173354; XM_006723920; XM_006726803; XM_011529474	-3.65	0.000274	down
DUSP1	NM_004417	-3.11	0.000016	down
SGK1	NM_001143676; NM_001143677; NM_001143678; NM_001291995; NM_005627; XM_011536071	-2.89	0.000006	down
SAT1	NM_002970; NR_027783	-2.71	0.000147	down
ZFP36	NM_003407	-2.68	0.000046	down
CTGF	NM_001901	-2.67	0.000008	down
NFKBIZ	NM_001005474; NM_031419	-2.59	0.000119	down
TAC1	NM_003182; NM_013996; NM_013997; NM_013998	-2.38	0.002457	down
ID2	NM_002166	-2.29	0.016465	down
IER2	NM_004907	-2.29	0.000075	down
RGS2	NM_002923	-2.2	0.000169	down
G0S2	NM_015714	-2.18	0.028634	down
THBS1	NM_003246; XM_011521970; XM_011521971; XR_931897	-2.18	0.00001	down
BHLHE40	NM_003670	-2.17	0.000085	down
KLF2	NM_016270	-2.07	0.0022	down
ZFAND5	NM_001102420; NM_001102421; NM_001278243; NM_001278244; NM_001278245; NM_006007	-2.06	0.012413	down
RHOB	NM_004040	-2.03	0.000044	down
JUNB	NM_002229	-2	0.000374	down
TRIB1	NM_001282985; NM_025195; XR_428373	-2	0.000268	down
ADAMTS1	NM_006988	-1.99	0.000157	down

TMEM107	NM_032354; NM_183065	-1.98	0.003219	down
C11orf96	NM_001145033	-1.93	0.009091	down
LIF	NM_001257135; NM_002309; XM_006724240; XM_011530172	-1.92	0.000061	down
IQGAP2	NM_001285460; NM_001285461; NM_001285462; NM_006633; XM_005248410; XM_005248414; XM_011543107; XM_011543108	-1.82	0.001088	down
MYC	NM_002467	-1.81	0.002517	down
PIM1	NM_001243186; NM_002648	-1.8	0.004771	down
MEST	NM_001253900; NM_001253901; NM_001253902; NM_002402; NM_177524; NM_177525; XM_011516222	-1.77	0.000005	down
DHRS3	NM_004753; XM_005263533; XM_005263534; XM_006711036	-1.75	0.002603	down
RCAN2	NM_001251973; NM_001251974; NM_005822; XM_006714948; XM_011514226	-1.75	0.000386	down
KLF4	NM_001314052; NM_004235; XM_005252305	-1.71	0.021515	down
SNORA12	NR_002954	-1.7	0.000183	down
CCNL1	NM_001308185; NM_020307; XM_005247647; XM_005247648; XM_005247649; XM_005247650;	-1.66	0.029876	down

	XM_005247651; XM_006713710; XM_011513011			
ANXA1	NM_000700; XM_011518608; XM_011518609	-1.65	0.019045	down
ATF3	NM_001030287; NM_001040619; NM_001206484; NM_001206485; NM_001206486; NM_001206488; NM_001674; NM_004024; XM_005273146; XM_011509579	-1.64	0.01664	down
EPGN	NM_001013442; NM_001270989; NM_001270990; NM_001270991; NM_001270992; NM_001270993; NR_073114; NR_073117; XM_005265666; XM_011531817	-1.63	0.000886	down
CREB5	NM_001011666; NM_004904; NM_182898; NM_182899; XM_005249906; XM_011515616; XM_011515617; XM_011515618	-1.63	0.002497	down
CPA4	NM_001163446; NM_016352	-1.61	0.011098	down
JUN	NM_002228	-1.6	0.000792	down
FAM46A	NM_017633	-1.6	0.005133	down
ZNF28	NM_001008801; NM_001277120; NM_006969; NM_199132; NR_036599; NR_036600;	-1.59	0.011836	down

	NR_102299;			
	NR_102300;			
	NR_102301;			
	XM_006723354;			
	XM_006723356;			
	XM_011527262;			
	XM_011527263			
CEBPD	NM_005195	-1.59	0.000528	down
	NM_001297592;			
	NM_001297594;			
	NM_015187;			
	XM_011513818;			
	XM_011513819;			
SEL1L3	XM_011513820	-1.59	0.024273	down
CXCL2	NM_002089	-1.59	0.00245	down
	NM_015715;			
	XM_011530203;			
	XM_011530204;			
	XM_011530205;			
PLA2G3	XR_937865	-1.58	0.036932	down
GADD45B	NM_015675	-1.58	0.000141	down
	NM_001008490;			
	NM_001160124;			
	NM_001160125;			
	NM_001300;			
KLF6	NR_027653	-1.57	0.000187	down
GDF15	NM_004864	-1.57	0.002575	down
	NM_001099743;			
	NM_001099744;			
	NM_001099745;			
	NM_001099746;			
	NM_001099747;			
	NM_001099748;			
	NM_001099749;			
	NM_001099750;			
	NM_001099751;			
	NM_001099752;			
	NM_001099753;			
	NM_001099754;			
	NM_001099755;			
	NM_001099756;			
	NM_017786;			
SYBU	XM_005250999;	-1.56	0.00068	down

	XM_005251000; XM_011517154			
EDIL3	NM_001278642; NM_005711; XM_011543088; XM_011543089	-1.56	0.001457	down
DOCK9	NM_001130048; NM_001130049; NM_001130050; NM_015296; XM_005254034; XM_005254035; XM_005254036; XM_006719922; XM_006719923; XM_006719924; XM_006719925; XM_006719926; XM_006719927; XM_006719928; XM_006719929; XM_006719930; XM_006719931; XM_006719932; XM_006719933; XM_006719935; XM_006719936; XM_006719937; XM_006719938; XM_006719941; XM_006719942; XM_011521063; XM_011521064	-1.56	0.004921	down
ARRDC3	NM_020801; XR_948281	-1.55	0.011918	down
KLF9	NM_001206	-1.54	0.024038	down
DXO	NM_005510; NM_178012; XM_006715005; XM_006715007; XM_006725474; XM_006725476; XM_006725688; XM_006725690;	-1.54	0.021426	down

	XM_006725898; XM_006725900; XM_006725985; XM_006725987; XM_006726078; XM_006726080; XM_011514571; XM_011547606; XR_926081; XR_926082; XR_952207; XR_952208; XR_952941; XR_952942; XR_953003; XR_953004; XR_953077; XR_953078			
ZNF469	NM_001127464; XM_011523386; XM_011523387; XM_011523388; XR_933463	-1.53	0.0082	down
PSAT1	NM_021154; NM_058179	-1.53	0.001464	down
BLZF1	NM_003666; XM_005245561; XM_011510092	-1.52	0.008234	down
SLC7A11	NM_014331; XM_011531800; XM_011531801; XM_011531802; XM_011531803; XM_011531804; XM_011531805	-1.51	0.000334	down
PLK2	NM_001252226; NM_006622	-1.51	0.005622	down
TAGLN	NM_001001522; NM_003186	-1.51	0.006653	down
TSC22D3	NM_001015881; NM_004089; NM_198057; XM_005262098; XM_005262099;	-1.51	0.000041	down

	XM_005262100;			
	XM_005262101;			
	XM_005262102;			
	XM_005262103;			
	XM_006724629;			
	XM_011530884			
	NM_001161708;			
SYNC	NM_030786	-1.51	0.000522	down
CYR61	NM_001554	-1.5	0.002574	down
PIP	NM_002652	-1.5	0.000199	down
NR0B1	NM_000475	-1.5	0.000461	down
	NM_001004688;			
	NM_001004689;			
	NM_001004690;			
	NM_001004691;			
OR2M1P	NR_002141	-1.5	0.031284	down
BAMBI	NM_012342	-1.5	0.021044	down
	NM_001017430;			
	NM_001017431;			
	NM_006743;			
	XM_011543938;			
RBM3	XM_011543939	-1.5	0.029936	down
	NM_001260474;			
	NM_001260475;			
	NM_001260476;			
	NM_001260477;			
	NM_018669;			
	NM_033661;			
	NR_048535;			
	XM_011529433;			
	XR_937429;			
	XR_937430;			
WDR4	XR_937431	1.5	0.031923	up
SLC24A3	NM_020689	1.51	0.0078	up
	NM_018518;			
	NM_182751;			
MCM10	XM_011519538	1.51	0.010484	up
	NM_001286503;			
	NM_001286504;			
	NM_001286505;			
	NM_006644;			
	XM_005266236;			
	XM_011534887;			
HSPH1	XM_011534888	1.51	0.01847	up

FGF7	NM_002009	1.51	0.000935	up
HIST1H2BF	NM_003522	1.52	0.004334	up
	NM_001201465;			
	NM_138966;			
	NM_138999;			
	NM_153181;			
	XM_005266774;			
	XM_005266776;			
	XM_011526218;			
	XR_935254;			
	XR_935255;			
	XR_935256;			
NETO1	XR_935257	1.53	0.00089	up
	NM_001789;			
	NM_201567;			
	XM_006713434;			
	XM_006713435;			
	XM_006713436;			
CDC25A	XM_011534316	1.54	0.018511	up
	NM_205855;			
FAM180A	XM_006715983	1.54	0.005888	up
	NM_014695;			
	NR_023380;			
	NR_036647;			
	NR_130142;			
	XM_005256877;			
	XM_006721607;			
	XM_011524088;			
CCDC144A	XM_011524089	1.54	0.042357	up
	NM_001254;			
	XM_011525541;			
CDC6	XM_011525542	1.55	0.005195	up
HBEGF	NM_001945	1.55	0.001331	up
	NR_027349;			
	NR_027350;			
	NR_029488;			
	NR_029489;			
	NR_029490;			
	NR_029492;			
MIR17HG	NR_029508	1.55	0.036166	up
	NM_001144073;			
	NM_012124;			
CHORDC1	XM_011542747;	1.55	0.005438	up

	XM_011542748; XM_011542749			
ITGA2	NM_002203; NR_073103; NR_073104; NR_073105; NR_073106; NR_073107	1.58	0.000286	up
FEN1	NM_004111	1.58	0.000061	up
SF3B3	NM_012426	1.59	0.010038	up
HSPA8	NM_006597; NM_153201; XM_011542798	1.6	0.000005	up
POSTN	NM_001135934; NM_001135935; NM_001135936; NM_001286665; NM_001286666; NM_001286667; NM_006475; XM_005266231; XM_005266232	1.62	0.007291	up
RGS4	NM_001102445; NM_001113380; NM_001113381; NM_005613	1.63	0.002293	up
FAM111B	NM_001142703; NM_001142704; NM_198947	1.64	0.001107	up
HIST1H2BK	NM_001312653; NM_080593	1.66	0.027174	up
MCM7	NM_001278595; NM_005916; NM_182776; XM_005250348	1.67	0.000986	up
CORIN	NM_001278585; NM_001278586; NM_006587	1.68	0.043444	up
STIP1	NM_001282652; NM_001282653; NM_006819	1.7	0.001893	up
SLCO4A1	NM_016354; XM_005260203; XM_011528791;	1.71	0.000049	up

	XM_011528792; XM_011528793; XM_011528794; XR_244115; XR_244116; XR_244117; XR_936522; XR_936523; XR_936524			
TM2D3	NM_001307960; NM_001308026; NM_025141; NM_078474; XM_005254980; XM_005254981	1.72	0.019979	up
HIST1H1D	NM_005320	1.75	0.039351	up
ESR2	NM_001040275; NM_001040276; NM_001214902; NM_001214903; NM_001271876; NM_001271877; NM_001291712; NM_001291723; NM_001437; NR_073496; NR_073497; XM_011536545; XM_011536546	1.75	0.006955	up
EWSR1	NM_001163285; NM_001163286; NM_001163287; NM_005243; NM_013986; XM_005261389; XM_005261390; XM_011529995; XM_011529996; XM_011529997; XM_011529998; XM_011529999; XM_011530000; XM_011530001; XM_011530002	1.75	0.040184	up

HIST1H2AL	NM_003511	1.76	0.004994	up
	NM_001242935;<br>NM_001308100;<br>NM_001308105;<br>NM_001308106;<br>NM_001308107;<br>NM_001308108;<br>NM_001308109;<br>NM_005460;<br>NR_131761;<br>NR_131762;<br>XM_005272138;<br>XM_005272139;<br>XM_006714734;<br>XM_011543736;<br>XM_011543737;<br>XM_011543738;<br>XM_011543739;<br>XM_011543740;<br>XM_011543741;<br>XM_011543742;<br>XM_011543743;<br>XM_011543744;<br>XM_011543745;<br>XM_011543746;<br>XM_011543747;<br>XM_011543748;<br>XM_011543749;<br>XM_011543750;			
SNCAIP	XM_011543751	1.77	0.030397	up
FZD8	NM_031866	1.86	0.000074	up
	NM_022455;<br>NM_172349;<br>XM_005265959;<br>XM_005265960;<br>XM_005265961;<br>XM_005265962;<br>XM_011534610;<br>XM_011534611;<br>XM_011534612;<br>XM_011534613;<br>XM_011534614;			
NSD1	XM_011534615;	1.87	0.021989	up

	XM_011534616; XM_011534617			
NTSR1	NM_002531; XM_011528827	1.91	0.004578	up
COL14A1	NM_021110; XM_005251059; XM_006716651	2	0.000988	up
FOXG1	NM_005249	2.1	0.000665	up
MAF	NM_001031804; NM_005360; XM_011523084	2.15	0.002975	up
RPS27A	NM_001135592; NM_001177413; NM_002954	2.17	0.005206	up
MMP1	NM_001145938; NM_002421	3.32	0.000008	up
